# A Randomized, Single‐Center, Double‐Blind, Controlled Case Study Evaluating Procedure Pairing of a Neurocosmetic Postprocedure Cream With Radiofrequency Microneedling for Facial Rejuvenation

**DOI:** 10.1111/jocd.16622

**Published:** 2024-10-10

**Authors:** McKenzie E. Maloney, Sofia Iglesia, Tatiana Kononov, Alisar S. Zahr, Michael H. Gold

**Affiliations:** ^1^ Medical College of Georgia Augusta University Augusta Georgia USA; ^2^ Revision Skincare Irving Texas USA; ^3^ Gold Skin Center Tennessee Clinical Research Center Nashville Tennessee USA

**Keywords:** antiaging, cosmeceuticals, microbiome, microneedling, neurocosmetic, postprocedure cream, radiofrequency, skincare

## Abstract

**Background:**

Radiofrequency (RF) microneedling produces patient discomfort which deters patients from completing the recommended treatment series.

**Objective:**

The primary objective was to determine the tolerability, safety, and efficacy of a neurocosmetic postprocedure cream post‐RF microneedling in reducing patient discomfort and enhancing recovery across the length of the study and, secondarily, to evaluate against a leading comparator. The third objective was to evaluate the efficacy of the neurocosmetic on self‐perceived improvement and objective grading.

**Materials and Methods:**

An Institutional Review Board (IRB) approved, fourteen‐day, randomized, single‐center, double‐blind, controlled clinical case study was conducted with 11 healthy female subjects, 6 randomized to the neurocosmetic and 5 to the comparator cell. Following a 7‐day washout period, subjects received RF microneedling (face and neck) and applied the postprocedure cream twice daily for 7 days. Objective and subjective tolerability, self‐assessments, and clinical photography were performed immediately postprocedure, 24 h, three and seven days following the procedure.

**Results:**

The neurocosmetic was tolerable and safe. Erythema and stinging immediately decreased postprocedure, postneurocosmetic application. After 24 h, 83% favorably agreed the neurocosmetic “reduced irritation on the skin post‐procedure,” and after 7 days, 100% favorably agreed “experience with the product was positive and I would be interested in returning for a second treatment.” The neurocosmetic reduced skin tone redness in the face and neck faster and to a greater degree when measured against a comparator.

**Conclusion:**

The neurocosmetic postprocedure cream improved patient discomfort and enhanced recovery when used immediately post‐RF microneedling and after 7 days.

**IRB Protocol Number:**

Pro00064211

## 1 Introduction

Aging is influenced by intrinsic and extrinsic factors. Amid aging, skin undergoes many changes, including increased degradation and reduced formation of collagen, increased oxidation and aggregation of macromolecules, solar elastosis, and mutagenesis [1, 2, 3]. These manifest as thinning, loss of elasticity, discoloration, wrinkling, and telangiectasias [3]. Ultraviolet radiation (UV) accelerates aging, and cumulative exposure results in coarse wrinkling, rough skin texture, as well as loss of elasticity [1, 2]. To combat aging, physicians recommend an armamentarium of topical treatments and procedures, including microneedling.

Microneedling, a minimally invasive, cosmetic, rejuvenation procedure, combats aging via cutaneous microwounds. Microwounds induce wound healing through the release of growth factors, leading to collagen and elastin production [4]. Histologically, the dermis thickens and tightens from the resulting production of hyaluronic acid, collagen, and elastin [5]. Coupling microneedling with radiofrequency (RF), 100 kHz–5 MHz, low‐frequency electromagnetic waves, increases efficacy by producing heat [6]. Heat promotes collagen and elastin formation and angiogenesis [7].

Microneedling creates microwounds, disrupting the skin's barrier function, which alters the innate skin microbiome. A complex relationship between the microbiome and wound healing exists [8]. Commensal bacteria aid wound healing by increasing granulation tissue deposition, collagen production, and angiogenesis [8]. Additionally, impaired barrier function increases transepidermal water loss (TEWL) immediately postmicroneedling and is directly proportional to skin dehydration [9]. Hydration status is considered a key factor for optimal healing [10]. Dry skin delays wound healing, resulting in poor cosmetic results [11].

The skin is a sensory organ, sending and receiving signals to and from the brain. This bidirectional relationship is mediated by signaling molecules [12]. Under stressful conditions, cellular proliferation and differentiation is impaired, decreasing lipid synthesis and barrier function and dysregulating healing biomarkers [12]. An integral mediator of the skin–brain axis is the transient receptor potential vanilloid 1 (TRPV1) receptor, a temperature‐sensitive calcium channel [13]. TRPV1, located on C and Aδ‐nerve fibers and keratinocytes, opens when exposed to heat greater than 42°C [14]. Calcium influx propagates an action potential, signaling the brain, resulting in the perception of pain. Antagonists of this receptor prevent signal propagation culminating in a net soothing effect, reducing stinging and burning, and thereby increasing levels of beta endorphin, skin's well‐being molecule [15]. TRPV1 antagonists have been found in neurocosmetic bioactives, topicals that leverage the skin–brain axis.

A neurocosmetic postprocedure cream was formulated as a breathable, water‐in‐oil delivery carrier composed of potent actives to modulate postprocedure relief as well as microbiome technology and hydrators to support compromised skin by regenerating the epidermal barrier. Specifically, TRPV1 antagonists, beta endorphin‐stimulating bioactives, and a bioavailable peptide were incorporated to ameliorate patient discomfort. Prebiotics, postbiotics, hyaluronic acid, ceramides, cholesterol, and phytosphingosine were formulated to assist in replenishing the skin's native microbiome to promote long‐term skin health. This neurocosmetic postprocedure cream was hypothesized to enhance recovery postprocedure and to increase the likelihood a patient will complete a recommended treatment series.

The first objective of this prospective case study was to evaluate the effect of the neurocosmetic on tolerability and safety parameters following RF microneedling when used immediately posttreatment and twice daily for one week. The secondary outcome was to compare and assess tolerability improvement of the neurocosmetic versus a leading comparator anhydrous cream. The third objective was to evaluate the efficacy of the neurocosmetic on self‐perceived improvement and objective grading.

## 2 Methods

Eleven subjects were enrolled in an IRB‐approved 14‐day randomized, single‐center, double‐blind, controlled clinical case study. Healthy females, aged 35–65, with Glogau Grade II or III were recruited. Subjects were excluded from the study if they were pregnant, breastfeeding, or planned to become pregnant during the study. The exclusion criteria also included having received a chemical peel, dermabrasion, or microneedling treatment in the previous six months; laser resurfacing (ablative, nonablative) in the previous twelve months; unwilling to refrain from pain medications postprocedure; unwilling to discontinue topical antiaging facial products for one week prior to study commencement or on prescription strength retinoids or skin lightening products within the previous two months.

A 7‐day washout period included twice‐daily use of a gentle foaming cleanser and basic facial moisturizer and a basic SPF 50 mineral sunscreen in the morning. At baseline, subjects were randomized into either the neurocosmetic cell (*n* = 6) or the comparator cell (*n* = 5).

Subjects visited the clinic at screening (Visit 1), after the 7‐day washout (Visit 2, baseline), one day postprocedure (Visit 3), three days postprocedure (Visit 4), and seven days postprocedure (Visit 5). Assessments were completed at all visits and pre‐ and postprocedure, including objective (investigator, board‐certified dermatologist) and subject tolerability, self‐assessment questionnaire, and clinical photography. Tolerability assessments evaluated the face and neck separately on a 4‐point scale (0 = none, 3 = severe), rating erythema, edema, dryness, and peeling, and stinging, tingling, itching, and burning, respectively. Investigator clinical grading, using the Glogau photoaging scale (Supporting Information Digital Content 1), was performed at Visit 1, Visit 2, and Visit 5. The Global Aesthetic Improvement Scale (GAIS) (Supporting Information Digital Content 1) was completed at Visit 5.

Morpheus8 (InMode, CA), a fractional bipolar radiofrequency microneedling device, was used at Visit 2. Prior to the procedure, patients were clinically evaluated, and clinical photographs were taken. Subjects were numbed 45 min prior to RF microneedling with topical numbing Benzocaine 20%/Lidocaine 6%/tetracaine 4%. Bony areas, periorbital, forehead, and chin were treated with 15 watts at a 2 mm needle depth on cycle mode. All subjects received 20 W at 3 mm depth on cycle mode on soft tissue, neck, and cheeks except one subject which received 18 watts due to tolerability.

Immediate postprocedure tolerability evaluations were completed followed by application of the neurocosmetic or comparator. This sequence took approximately 20 min. Evaluations, clinical photography, and self‐assessments were completed postprocedure, postproduct application.

Three‐dimensional images of the face and neck were taken with QuantifiCare LifeViz Mini (Quantificare, GA), and the skin assessment tool generated red map facial images. Standardized templates were created for the forehead, neck, cheek, and full face. ImageJ (National Institutes of Health, MD), an open‐source software that processes and analyses images [16], objectively measured the pixel intensity of redness of these standardized templates. A maximum pixel intensity of 255 represents complete skin tone evenness (brightness) and a pixel intensity of 140 or less represents maximum skin tone redness.

Satisfaction with the efficacy and aesthetics of the products were evaluated with a self‐assessment questionnaire (Supporting Information Digital Content 1), which was analyzed based on subjects scoring a 4 or 5 (favorably agreed).

### 2.1 Statistical Analysis

Statistical analyses were conducted in R Statistical Software (v4.1.2; R Core Team 2021). Postprocedure was set as the baseline for quantitative comparisons as it represented the most severe condition. Due to a nonnormal distribution, median scores were used in the Wilcoxon signed‐rank test for one‐sample analysis and the Mann–Whitney *U* tests for the two‐sample analysis (between cells). The significance level was set to 90% confidence with *p < 0.10 due to the small sample size of this study.

## 3 Results

The average age of subjects in the neurocosmetic and comparator cells was similar, 48.2 years versus 50.0 years, respectively, Table 1. Randomized subjects had Fitzpatrick Skin Types II–IV in the neurocosmetic cell and Fitzpatrick Skin Type II–III in the comparator cell. The majority of subjects, 63.6%, were Fitzpatrick Skin Type III followed by Type II (27.3%) and Type I (9.1%). None of the subjects previously received RF microneedling.

**TABLE 1:** Demographics of subjects who completed the study.

	Neurocosmetic PPC cell	Comparator anhydrous cream cell	Total
Sample size	6	5	11
Average age	48.2	50.0	49.0
Fitzpatrick skin type			
II	1	2	3
III	4	3	7
IV	1	0	1

### 3.1 Tolerability and Safety Assessment

Postprocedure, erythema on the face and neck was the only objective tolerability parameter with a majority of nonzero values at baseline in both cells. Subgroup analysis was performed in both cells with nonzero erythema scores. The neurocosmetic and comparator had similar erythema results on postprocedure days 1, 3, and 7 for the face and neck with a 100% median improvement (**p < 0.05); however, there was one notable difference between the neurocosmetic and comparator. Immediately posttreatment, postneurocosmetic 40% and 50% of subjects improved in facial and neck erythema, respectively, versus 0% of subjects improved in the comparator cell for both face and neck. At Visit 5, 100% of subjects improved in facial and neck erythema, indicating the neurocosmetic was tolerable and enhanced recovery postprocedure.

Postprocedure, stinging was the predominant tolerability parameter in both cells with nonzero baseline values for face and neck. Fifty percent of subjects scored nonzero values for stinging postprocedure on the face and 33% scored nonzero values for stinging on neck in the neurocosmetic cell. Sixty percent of subjects in the comparator cell scored nonzero values for stinging on face and neck postprocedure (baseline).

Subgroup analysis was performed on stinging in both cells on nonzero stinging values. Sixty‐seven percent improved in stinging immediately after application of both the neurocosmetic and comparator on the face. From Visit 3 to Visit 5, 100% of subjects improved in stinging when using the neurocosmetic for 7 days with a 100% median improvement (*p* = 0.20). Immediately post‐RF microneedling and postproduct application, there was a 75% median percent improvement in stinging of the neck in the neurocosmetic cell (*p* = 0.21) versus 50% median percent improvement in the comparator (*p* = 0.18). Additionally, from Visit 3 to Visit 5, 100% median improvement across the remaining visits in stinging (*p* = 0.21) in the neurocosmetic cell. No serious adverse events occurred.

### 3.2 Clinical Photography

**FIGURE 1:**
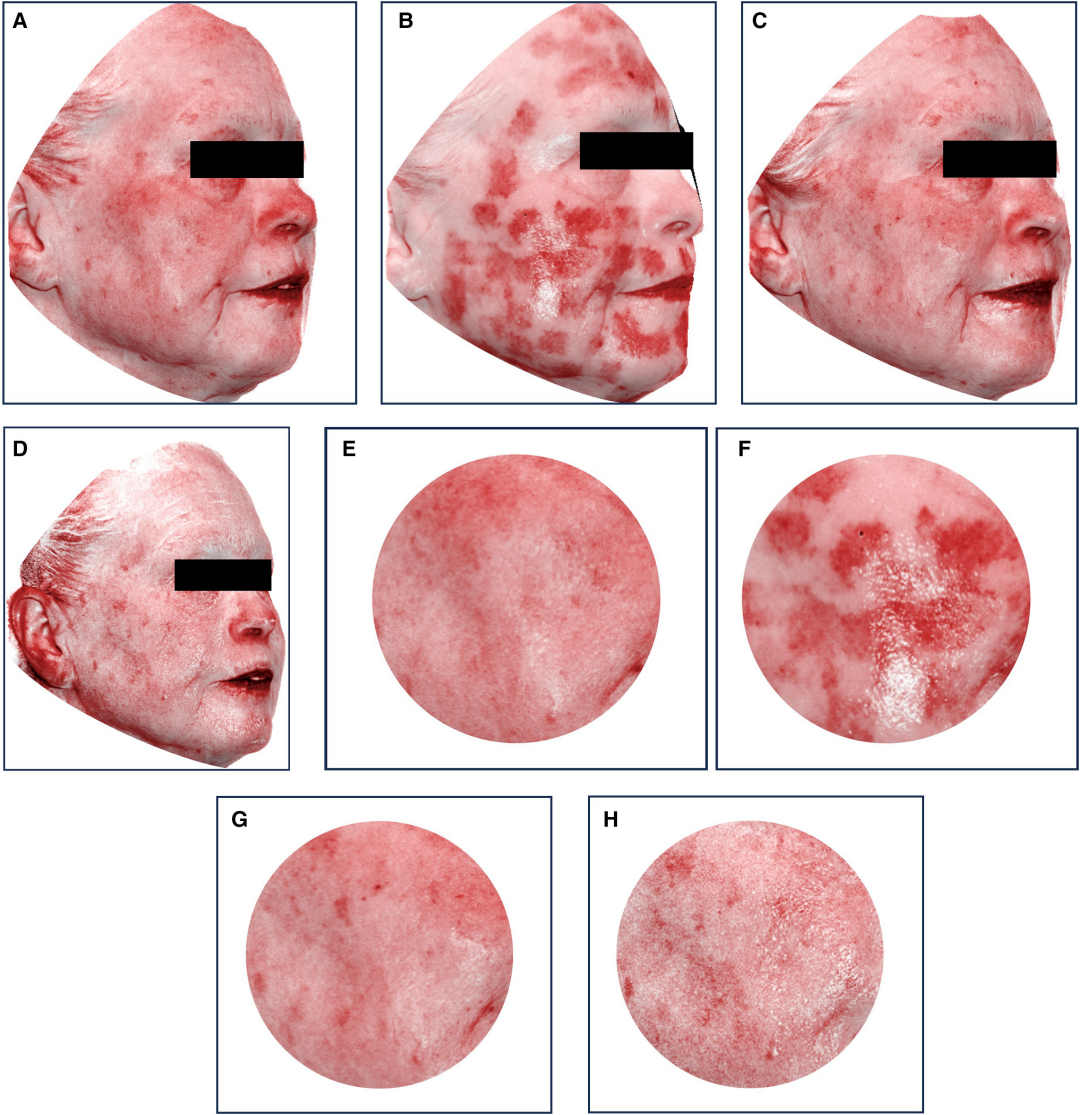
Representative 3D photographs (red map) of a 63‐year‐old female with Fitzpatrick Skin Type II using the neurocosmetic. (A) Preprocedure, (B) postprocedure, postproduct application, (C) 24‐h postprocedure (V3), (D) 7 days postprocedure (V5). Right cheek templates (F–H) are shown at the same time points.

**FIGURE 2:**
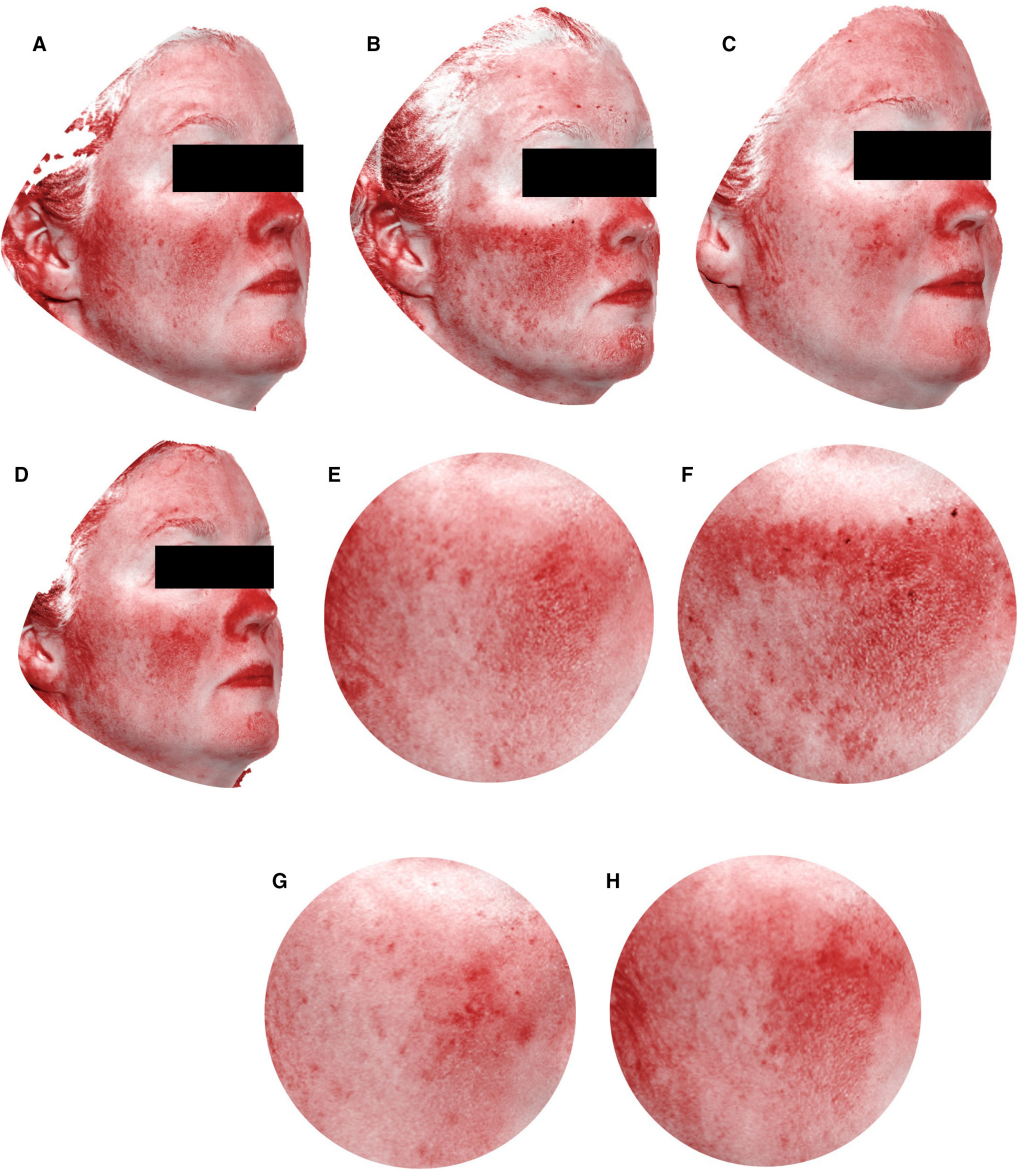
Representative 3D photographs (red map) of a 42‐year‐old female with Fitzpatrick Skin Type II using the comparator. (A) Preprocedure, (B) postprocedure, postproduct application, (C) 24‐h postprocedure (V3), (D) 7 days postprocedure (V5). Right cheek templates (F–H) are shown at the same time points.

Representative subjects from the neurocosmetic and the comparator cells are shown in Figures 1 and 2, respectively. In Figure 1, there was a 4% and 9% reduction in skin tone redness (increased skin brightness) of the right cheek after Visit 3 and Visit 5 compared to postprocedure postneurocosmetic, respectively. In Figure 2, there was an 8% reduction in skin tone redness (increased skin brightness) of the right cheek after Visit 3% and 1% increase in skin tone redness (decreased skin brightness) of the right cheek after Visit 5 compared to postprocedure, postcomparator, respectively.

**FIGURE 3:**
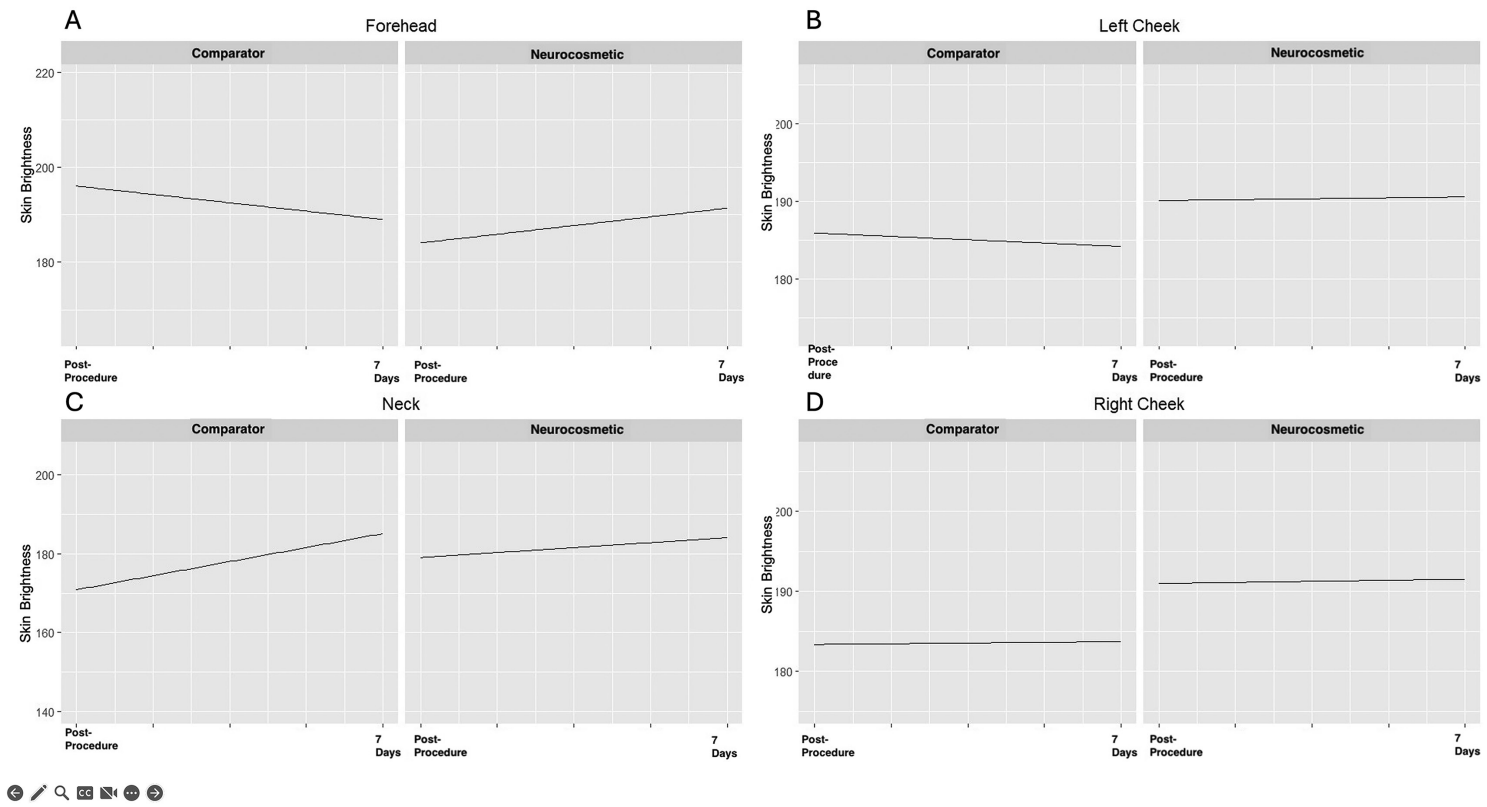
Average pixel analysis measuring skin brightness for the forehead, neck, left cheek, and right cheek over time from post‐procedure post‐product application to 7 days post‐procedure. Positive trendlines indicate decreased redness, and negative trendlines indicate increased redness.

Pixel intensity for skin tone redness measured by ImageJ of the face and neck for both the neurocosmetic and comparator treated cells were plotted at each postprocedure timepoint and for each subject within the treatment cell, Figure 3. The neurocosmetic cell demonstrated a positive trend of decreased skin tone redness over time, and the comparator cell demonstrated a negative trend of increased skin tone redness over time for the forehead and left cheek. On the neck, the neurocosmetic and the comparator performed equally well, demonstrating a reduction in skin tone redness. Evaluating combined treatment areas, the neurocosmetic and comparator cells demonstrated overall positive trends. The slope of the trend line for the neurocosmetic was larger than the comparator, thereby, giving visual evidence that skin tone redness was reduced at a higher rate. Furthermore, the neurocosmetic was three and two times more effective at reducing skin tone redness after 24 h and 7 days compared to the comparator, respectively. This observation indicates the neurocosmetic postprocedure cream enhances recovery faster compared to the comparator.

### 3.3 Clinical Efficacy Assessment

Preprocedure, in the neurocosmetic cell, 50% had Glogau Type II and 50% Type III scores corresponding to wrinkles in motion and at rest, respectively. At Visit 5, 50% had Type II and 50% Type III scores. The neurocosmetic did not negatively impact wrinkles and skin discoloration. The Visit 5 GAIS analysis showed 67% of the neurocosmetic cells improved, and 33% were much improved.

### 3.4 Self‐Assessment Questionnaire

**FIGURE 4:**
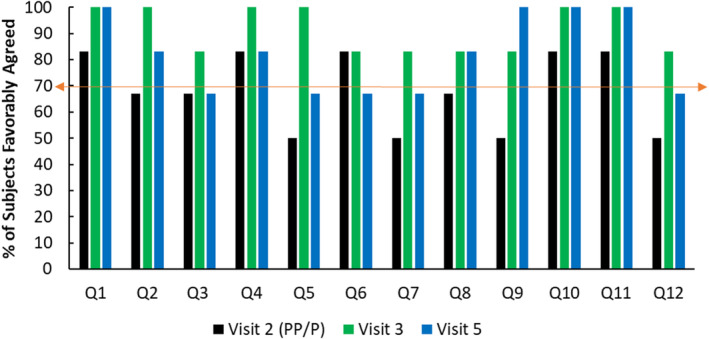
Self‐assessment questionnaire results from visits 2, 3, and 5.

Postprocedure, postneurocosmetic application, 83% favorably agreed that the product was gentle, immediately moisturized, provided nourishing hydration, comforted the skin postprocedure, and reduced irritation postprocedure (Figure 4).

On postprocedure day 1, the following percent of subjects favorably agreed.

- 100%: The product was gentle, calming, provided nourishing hydration, immediately moisturized, comforted skin postprocedure, relieved discomfort, and that their skin looked and felt smooth.
- 83%: The product smoothed burning, reduced irritation postprocedure, reduced redness, alleviated dry skin, protected their skin postprocedure, relieved dry, damaged skin, improved skin texture and appearance, and eased recovery time postprocedure.

On postprocedure day 7, the following percent of subjects favorably agreed.

- 100%: “The experience with product and procedure was positive and I would be interested in returning for a second treatment.”
- 100%: The product was gentle, protected their skin postprocedure, provided nourishing hydration, immediately moisturized, improved the appearance of their skin, and that the product eased recovery time postprocedure.
- 83%: The product calmed, comforted skin postprocedure, relieved dry, damaged skin, improved skin texture, enhanced and accelerated healing time, helped skin recovery faster, and that their skin looked and felt smooth.

## 4 Discussion

As patients increasingly seek aesthetic rejuvenation treatments such as RF microneedling, a postprocedure cosmeceutical that improves posttreatment tolerability, to ensure completion of a treatment series, is an unmet need. The evaluated neurocosmetic postprocedure cream is a semi‐occlusive, breathable, reverse emulsion delivery carrier, which provides relief posttreatment and enhances recovery by supporting the regeneration of the epidermal barrier and skin microbiome. The neurocosmetic is formulated with bioactives, *Albatrellus confluens* [17], *Helichrysum italicum* extract [18], *Daucus carota sativa* root extract [19], and *Opuntia ficus‐indica callus* [20] extract shown to support sensations of relaxation, calmness, and comfort, allowing for enhanced recovery, and increasing the likelihood patients return for multiple treatments.

Erythema, determined by the investigator, significantly improved 24 h following application of the neurocosmetic postprocedure cream, post‐RF microneedling. Erythema is a frequently cited side effect of RF microneedling which lasts from 12 h to 5 days [21]. Other common side effects include edema, pain, and bleeding, and less commonly reported, xerosis and scaling [21]. Side effects may deter subjects from completing the recommended treatment series. The neurocosmetic alleviated erythema faster and to a greater degree than the comparator while providing subjects with nourishing hydration, soothing dry skin, and reducing the impact of common adverse effects.

The neurocosmetic minimized pain, heat sensations, stinging, and erythema, but did not necessarily reduce inflammation. Neurocosmetics target keratinocytes and cutaneous nerve endings, desensitizing them to cold, heat, pain, pressure, and itch, providing skin soothing, reducing itchiness and erythema, and impacting patients' well‐being [16, 22]. The skin and brain share embryonic origins, the ectoderm, and are interconnected by nerves, communicating via neurotransmitters [23]. Neurocosmetics increase beta‐endorphins in keratinocytes and sensory neurons, improving keratinocyte differentiation, barrier repair, and wound healing [22]. When skin receives a stress signal (e.g., heat), the pain cycle is activated with upregulation of nociceptors (TRPV1). The neurocosmetic was formulated to interrupt this cycle by increasing beta‐endorphins, reducing the concentration of calcitonin gene‐related peptide (CGRP), and blocking TRPV1, thus reducing pain signaling [22]. CGRP is a small protein found in sensory nerves involved in pain transmission, and concentrations increase during skin injury [24]. Blocking TRPV1 decreases nociceptor sensitivity and nourishes the skin's native microbiome, promoting skin barrier repair [25]. Further, the neurocosmetic contains potent antioxidants including tetrahexyldecyl (THD) ascorbate, ceramides, and prebiotic inulin which assist in recovery by reducing procedural downtime.

The neurocosmetic objectively reduced erythema faster and to a greater degree than the comparator, suggesting the neurocosmetic delivered pain relief to distressed skin in the critical days following procedure by regenerating the epidermal barrier and native microbiome. Following RF microneedling there is a natural elevation in TEWL [9]. The neurocosmetic is a reverse emulsion, semi‐occlusive, allowing the skin to breathe while reducing TEWL. In contrast to the comparator, an anhydrous formulation that sits on the skin trapping moisture but is not always optimal for skin health, the neurocosmetic is breathable, providing skin nourishment, protection, and moisturization, especially in critical times postprocedure.

The neurocosmetic increased subject tolerability as the majority favorably agreed that the product was beneficial. All subjects agreed that “the experience with the product and procedure was positive and I would be interested in returning for a second treatment.” Therefore, this neurocosmetic effectively increases tolerability, increasing the likelihood that subjects will complete the recommended treatment series.

This study was limited by the small sample size inherent to this pilot case study. Therefore, the probability of a Type II error is more likely, and differences between the two cells would have been statistically significant with a larger sample size as the neurocosmetic was trending toward greater tolerability and efficacy for many measures compared to the comparator. Additionally, subjects had zero values for many investigator tolerability parameters (edema, dryness, itching, and burning). Therefore, the products could not improve upon a zero baseline, and thus, the product's benefits may be underreported.

## Author Contributions

All authors have read and approved the final manuscript. M.H.G. performed the research. A.S.Z., S.I., and T.K. designed the research study. M.E.M., S.I. and A.S.Z. wrote the paper.

## Conflicts of Interest

McKenzie E. Maloney, Tatiana Kononov, and Michael Gold are consultants at Revision Skincare. Sofia Iglesia and Alisar Zahr are full‐time Revision Skincare employees. Michael Gold is the Editor‐in‐Chief of the *Journal of Cosmetic Dermatology*.

## Supporting information


Supporting Information Digital Content 1.

## Data Availability

The data that support the findings of this study are available from the corresponding author upon reasonable request.
