# Supplementary material for: A Randomized, Single‐Center, Double‐Blind, Controlled Case Study Evaluating Procedure Pairing of a Neurocosmetic Postprocedure Cream With Radiofrequency Microneedling for Facial Rejuvenation
Source: J Cosmet Dermatol. 2024 Oct 10;23(12):4077–84. doi: 10.1111/jocd.16622 (PMC11626329; doi:10.1111/jocd.16622)
Supplement: Supplementary file 1 — Supporting Information Digital Content 1. [file JOCD-23--s001.docx]

**Supplementary Digital Content 1.** Clinical Grading Scales

| Glogau Grading Scale | |
| --- | --- |
| Grade I | No wrinkles. Minimal to no discoloration or wrinkling. No keratoses. |
| Grade II | Wrinkles in motion. Wrinkling I skin with movement. Slight lines near the mouth and eyes. No keratoses. |
| Grade III | Wrinkles at rest. Visible wrinkles all the time. Noticeable discolorations. Visible keratoses. |
| Grade IV | Only wrinkles. Wrinkles throughout (make-up appears to cake and crack when applied). Grey or yellow discoloration of the skin. History of prior skin cancer. |
| Global Aesthetic Improvement Scale (GAIS) | |
| Very much improved | Optimal cosmetic result for the treatment in this patient. |
| Much improved | Marked improvement in appearance but not completely optimal for this patient. A touch-up would slightly improve the result. |
| Improved | Obvious improvement in appearance from the initial condition, but a touch-up or re-treatment is indicated. |
| No change | The appearance is the same as than the original condition. |
| Worse | The appearance is worse than the original condition. |

**Supplementary Digital Content 2.** Self-Assessment Questionnaire. Questions 1-15 were completed at Visit 2 (V2) post-procedure post-product. Questions 1-20 were completed at Visit 3 (V3) one day post-procedure. Questions 1-25 were completed at Visit 5 (V5) seven days post-procedure.

| 1. The product was gentle on my skin. |
| --- |
| 1. The product calmed my skin. |
| 1. The product soothed my skin (from burning sensation) |
| 1. The product comforted my skin post-procedure. |
| 1. The product helped reduce the feeling of warmth/heat on my skin. |
| 1. The product relieved discomfort of skin. |
| 1. The product reduced irritation on the skin post-procedure. |
| 1. The product reduced redness on my skin. |
| 1. The product provided relief to dry, damaged skin. |
| 1. The product relieved itchiness of my skin. |
| 1. The product helps to protect my skin post-procedure. |
| 1. The product provided nourishing hydration to my skin. |
| 1. The product moisturizes my skin immediately. |
| 1. The product alleviated my dry skin. |
| 1. The product helped alleviate pain and discomfort post-procedure. |
| 1. The product reduced swelling (inflammation). |
| 1. The product helped improve the texture of my skin. |
| 1. My skin looks and feels smooth (texture of skin). |
| 1. The appearance of my skin is improved. |
| 1. The product eased my recovery time post-procedure. |
| 1. The product enhanced and accelerated my healing time. |
| 1. The product helped my skin recover faster. |
| 1. I like the product aesthetics (appearance, smell, feel). |
| 1. The appearance of skin is improved (glow to my skin and skin tone). |
| 1. The experience with product and procedure was positive and I would be interested in returning for a second treatment. |
